# Limited Lateral Transport Bias During Export of Sea Surface Temperature Proxy Carriers in the Mediterranean Sea

**DOI:** 10.1029/2021GL096859

**Published:** 2022-02-23

**Authors:** Addison Rice, Peter D. Nooteboom, Erik van Sebille, Francien Peterse, Martin Ziegler, Appy Sluijs

**Affiliations:** ^1^ Department of Earth Sciences Utrecht University Utrecht The Netherlands; ^2^ Department of Physics IMAU Utrecht University Utrecht The Netherlands; ^3^ Centre for Complex Systems Studies Utrecht University Utrecht The Netherlands

## Abstract

Some lipid‐biomarker‐based sea surface temperature (SST) proxies applied in the modern Mediterranean Sea exhibit large offsets from expected values, generating uncertainties in climate reconstructions. Lateral transport of proxy carriers along ocean currents prior to burial can contribute to this offset between reconstructed and expected SSTs. We perform virtual particle tracking experiments to simulate transport prior to and during sinking and derive a quantitative estimate of transport bias for alkenones and glycerol dibiphytanyl glycerol tetraethers (GDGTs), which form the basis of the U^K’^_37_ and TEX_86_ paleothermometers, respectively. We use a simple 30‐day surface advection scenario and sinking speeds appropriate for the export of various proxy carriers (6, 12, 25, 50, 100, 250, 500, and 1000 md^−1^). For the assessed scenarios, lateral transport bias is generally small (always <0.85°C) within the Mediterranean Sea and does not substantially contribute to uncertainties in U^K’^_37_‐ or TEX_86_‐based SSTs.

## Key Points

- Simulated lateral transport during export of biomarker lipids does not impact U^K'^_37_ or TEX_86_ sea surface temperature reconstructions in the Mediterranean Sea

## 1 Introduction

Many paleoclimate reconstructions rely on geochemical proxies to determine past environmental changes. However, in Mediterranean Sea surface sediments, certain sea surface temperature (SST) proxies often yield values that are offset from the expected SST (Grauel et al., 2013; Kim et al., 2015; Leider et al., 2010; Tierney & Tingley, 2014, 2018). Two common SST proxies that exhibit large offsets from mean annual SSTs in this region are U^K’^_37_ (Prahl et al., 1988), and TEX_86_ (Schouten et al., 2002). The U^K’^_37_ paleothermometer is based on the degree of unsaturation of C_37_ alkenones produced by haptophyte algae such as coccolithophorids, where more di‐unsaturated C_37_ alkenones are produced relative to tri‐unsaturated C_37_ alkenones at higher temperatures. The TEX_86_ paleothermometer is based on the relative abundance of different glycerol dibiphytanyl glycerol tetraethers (GDGTs) produced largely by Thaumarchaeota, a group of marine archaea. Higher temperatures result in a larger average number of rings in the GDGT assemblage. In the Mediterranean Sea, U^K’^_37_‐based SSTs in surface sediments are generally 2–4°C colder than mean annual values (Tierney & Tingley, 2018), whereas TEX_86_‐based SSTs are generally 2–6°C warmer (Kim et al., 2015). Sediment core studies have shown that these proxies exhibit differences in temperature amplitudes across climate transitions (Castañeda et al., 2010; Grauel et al., 2013) and sapropel events (Menzel et al., 2006; Polik et al., 2018). High residuals in Mediterranean Sea samples in surface sediment U^K’^_37_ and TEX_86_ calibration studies have been attributed primarily to seasonal production of alkenones (Sicre et al., 1999; Ternois et al., 1997; Tierney & Tingley, 2018) and GDGT contributions of deep‐water dwelling Thaumarchaeota (Besseling et al., 2019; Kim et al., 2015), respectively (see Text S1 in Supporting Information S1). However, the role of ocean currents in transporting particles during export has not been fully assessed. In the Mediterranean, surface flow is generally from west to east, bringing colder and fresher Modified Atlantic Water further into the basin (Roussenov et al., 1995). Temperature and salinity gradients largely follow this direction, with increasing surface temperature and salinity from west to east. Because the temperature gradient follows the direction of flow, it is possible that lateral transport could result in a consistent cold bias in proxies that originate near the surface, including U^K’^_37_. Subsurface waters follow the opposite direction; Levantine Intermediate Water is produced in the Eastern Mediterranean and flows westward. This water mass is associated with GDGT‐producing archaea (Besseling et al., 2019; Kim et al., 2016), and could transport GDGTs from east to west, possibly resulting in a warm bias in the TEX_86_ paleothermometer.

Previous studies using Lagrangian particle tracing experiments have shown that transport by ocean currents can result in large offsets in inorganic SST proxies, both during the organism's life and export of the proxy carrier to the sea floor (Nooteboom et al., 2019; van Sebille et al., 2015). Dämmer et al. (2020) simulated trajectories of living foraminifera in the Mediterranean Sea and noted that the temperature and salinity history recorded in their tests during their life may differ from the sea surface conditions at the location of their burial. However, the mean temperature experienced along the virtual foraminifer's trajectory is similar to the SST above their burial location. Slow‐sinking dinoflagellate cysts may also exhibit large offsets in SST between the location of their formation and the location of their burial (Nooteboom et al., 2019). Lateral transport by ocean currents is occasionally posited as a source of bias in biomarker‐based proxies as well. For example, Benthien and Müller (2000) suggested that lateral transport of alkenones could impact the U^K’^_37_ paleothermometer in the western South Atlantic, where sinking particles are subject to strong surface currents. Similarly, Kim et al. (2009) found that alkenones in sediments from the South East Indian Ridge originated from distant sources, while GDGTs represent a local signal. However, a sediment trap study from the Mozambique Channel comparing eddy variability with organic proxies suggests that lateral transport in the water column is unlikely to greatly impact distributions of alkenones and GDGTs (Fallet et al., 2011). Alkenones and GDGTs are produced by different organisms, possibly in different seasons, and at different depths. Furthermore, sediment trap studies exhibit differences in alkenone and GDGT sinking speeds, with GDGT‐carrying particles sinking more slowly than those carrying alkenones, likely related to differences in export mechanisms (Fallet et al., 2011, 2012; Mollenhauer et al., 2015; Richey & Tierney, 2016). These differences could result in different impacts of lateral transport on proxy‐based temperatures.

Here, we assess the occurrence of lateral transport bias during export in the Mediterranean Sea by simulating the trajectories of sinking particles through the water column and in the surface ocean and comparing surface sediment proxy offsets to the simulated transport bias.

## 2 Methods

### 2.1 Simulation Setup

The simulation uses Parcels version 2.1.6 (Delandmeter & van Sebille, 2019) to advect particles through the model. Simulations release virtual particles from the ocean floor and backtrack them to their location at a 30 m water depth for sinking scenarios, while the surface transport scenario tracks virtual particles at a constant 30 m water depth. Particles move according to the flow field from the Nucleus for European Modeling of the Ocean (NEMO; Madec, 2016; Storkey et al., 2010; Uotila et al., 2017), which has a 5‐daily and 1/12° resolution. This model was chosen because it is sufficient for resolving mesoscale eddies in low‐to mid‐latitude regions including the Mediterranean Sea, which is recommended for Lagrangian particle tracking experiments (Nooteboom et al., 2020; Qin et al., 2014). The particle trajectories were integrated with a Runge‐Kutta 4 scheme with a time step of 10 min, and no additional diffusion was added to the trajectories. The assigned sinking speed is added to the vertical movement in the flow field. Simulated particles were released every 5 days during model year 2009, chosen to allow particles to move backwards in time through the available flow field. Because surface sediments generally consist of decades or centuries of accumulated material, any year that is representative of typical circulation patterns should yield representative simulations. Virtual particles reached the 30 m water depth during model years 2007–2009, depending on the sinking speed and water depth at the site, where trajectory endpoint SSTs were recorded. The locations of virtual particles were also recorded at 150 m depth.

### 2.2 Surface Sediment Starting Locations

For comparison with proxy results, surface sediment locations compiled in calibration studies for U^K’^_37_ (Tierney & Tingley, 2018) and TEX_86_ (Kim et al., 2015) serve as startng ponts for simulated trajectories. Locations with less than 30 m water depth were removed, resulting in a data set of 91 and 195 sites for U^K’^_37_ and TEX_86_, respectively, with water depths up to 3,577 m. These sites were consolidated such that sites within 10 km were considered as one location, resulting in a total of 189 locations that serve as starting points in the simulation. Mean annual SSTs in this data set range from about 15 to 25°C, with strong seasonal variability of 4–14°C. In general, mean annual SST increases from west to east and from north to south. Winter SSTs exhibit a strong north‐south gradient, while summer SSTs exhibit more spatial variability (Pastor et al., 2018). For spatial analysis, the trajectory data set was binned by the subbasin (Figure S1 in Supporting Information S1).

### 2.3 Sinking Speeds

Sediment trap studies can constrain sinking speeds for different export mechanisms by providing an average rate of sinking between two water depths, either between the ocean surface and the sediment trap or between sediment traps set at multiple depths (Fischer & Karakaş, 2009). Sediment trap studies assessing sinking speeds of haptophyte algae remains in the Mediterranean Sea and of alkenone‐ and GDGT‐carrying particles are reviewed here.

Alkenones can be exported from the surface ocean via several export mechanisms, including fecal pellets (Thomsen et al., 1998), aggregates, coccospheres, and coccoliths. Turner (2002) reviewed sinking rates of marine snow and fecal pellets, finding reported sinking speeds of 16–368 md^−1^ for marine snow and 5–2700 md^−1^ for fecal pellets, with large variations between ecological groups. Sinking speed estimates from sediment trap data in the oligotrophic eastern Mediterranean are 100 md^−1^ for coccospheres and 21 md^−1^ for coccoliths (Ziveri et al., 2000). In the Cretan Sea, coccoliths sink at about 33 md^−1^ (Triantaphyllou et al., 2004). Sediment trap studies examining U^K’^_37_ values sometimes note an offset between the seasonal SST and the U^K’^_37_‐based SST, allowing researchers to calculate an average sinking speed between the surface and the depth of the sediment trap. Mollenhauer et al. (2015) suggest a sinking speed of 14–59 md^−1^ near Cape Blanc, while Richey and Tierney (2016) calculate 34 md^−1^ in the Gulf of Mexico. Others note a lack of seasonal signals, indicating slow sinking speeds (Fallet et al., 2011, 2012). In a sinking velocity sediment trap study directly measuring the velocity of particles as they enter the trap, Wakeham et al. (2009) observed a bimodal distribution of sinking speeds for alkenone‐carrying particles in the western Mediterranean. Some are fast‐sinking (>49 md^−1^), but most of the flux of alkenones is associated with particles that sink at intermediate speeds (11–49 md^−1^).

Export mechanisms for GDGTs are poorly understood but presumably include aggregates and fecal pellets. Sediment traps again constrain the sinking speeds appropriate to describe GDGT‐carrying particles. Based on seasonal SSTs, Mollenhauer et al. (2015) calculate a sinking speed of 9–17 md^−1^ near Cape Blanc, while Yamamoto et al. (2012) find a sinking speed of at least 260 md^−1^ in the western North Pacific, and Wuchter et al. (2006) calculate 25–75 md^−1^ speeds in the Arabian Sea. Many studies note a lack of seasonality in the TEX_86_ signal in sediment traps, possibly indicating slow sinking speeds obscuring seasonal temperature changes (Chen et al., 2016; Fallet et al., 2011, 2012; Richey & Tierney, 2016).

Although sediment traps can help to constrain the average sinking speeds between two water depths, sinking speeds increase with water depth (Berelson, 2001; Fischer & Karakaş, 2009). Furthermore, small suspended particles may take time to aggregate before sinking or prior to grazing, possibly allowing transport by surface currents prior to aggregation and rapid export. Nooteboom et al. (2019) tested two scenarios in which sinking speed increased with depth (6 to 45 md^−1^ and 6 to 65 md^−1^), and found that results for transport distance and SST offsets were similar to the 6 and 11 md^−1^ sinking speeds also tested in their study, suggesting that transport near the surface is most important for assessing bias due to lateral transport. We, here explore this process with a scenario in which particles are backtracked at a constant water depth of 30 m for 30 days. This scenario simulates small particles that are advected by surface currents, aggregate above the burial site, and sink quickly. It also approximates increasing sinking speeds with depth, since most lateral transports occur in the surface ocean prior to sinking. To assess the importance of different export modes, the simulation is run with a range of approximately doubling sinking speeds (6, 12, 25, 50, 100, 250, 500, and 1000 md^−1^) to describe the alkenone and GDGT export. A sinking speed of 1000 md^−1^ represents fast‐sinking aggregates and fecal pellets and is representative of conditions immediately above the surface sediment site. Results from the 1000 md^−1^ trajectories are used as a comparison point for the trajectories with slower sinking speeds, which represent the export of small or low‐density particles. By using the 1000 md^−1^ trajectory results, we directly compare whether fast‐sinking or slow‐sinking export modes better represent the proxy signal accumulated in surface sediments.

### 2.4 Calculation of Transport Bias and Comparison With Proxy Data

**Figure 1:**
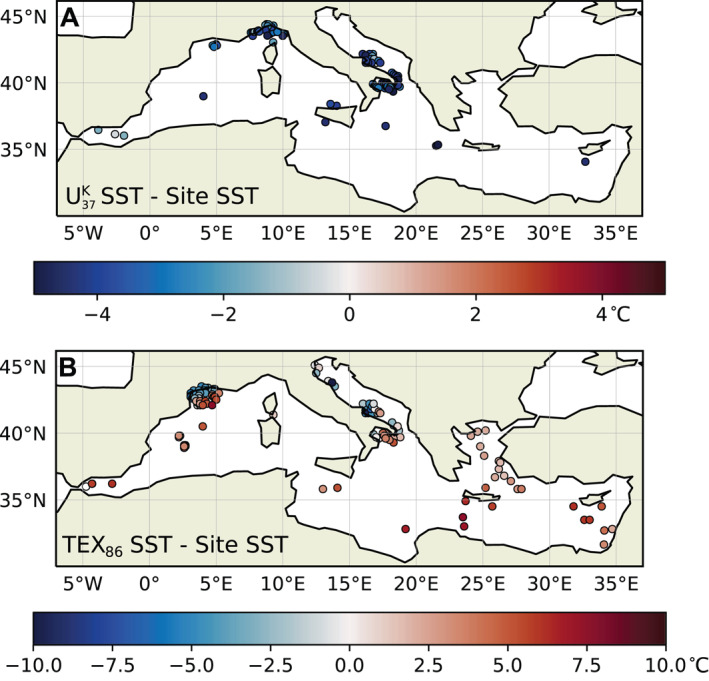
Differences between proxy‐based sea surface temperatures (SSTs) (a) U^K’^_37_ and (b) TEX_86_ and 1000 md^−1^ SSTs. Positive (red) values indicate that the proxy overestimates SST.

Results from simulated trajectories are summarized by the sediment location from which particles have been backtracked. For each burial location and sinking speed, the mean simulated distance between trajectory endpoints in the surface ocean and burial locations (trajectory starting points) was calculated. For calculation of transport bias and proxy bias, the mean of SSTs recorded by the simulation at the 1000 md^−1^ trajectory endpoints, corresponding to fast‐sinking aggregates, is considered to be representative of site conditions (SST_site_). Proxy‐based temperatures (SST_proxy_) were calculated using the Prahl et al. (1988) calibration for U^K’^_37_ and the Kim et al. (2010) calibration for TEX_86_. Proxy offsets for U^K’^_37_ and TEX_86_ were calculated as SST_proxy_–SST_site_ (Figure 1).

Mean simulated transport bias is calculated as the difference between the mean of SSTs recorded in the surface ocean at the trajectory endpoints (SST_end_) for a particular export scenario relative to the mean of SSTs recorded at the 1000 md^−1^ trajectory endpoints (SST_site_). The mean magnitude of transport bias for a given sinking speed is calculated as the mean of the absolute value of mean transport bias recorded at each burial location. Transport bias is considered to be negligible if it is smaller than proxy analytical uncertainty. Modern reproducibility values for U^K’^_37_ are in the range of ±0.002 units (about ±0.1°C; Tierney & Tingley, 2018), while older studies note report higher analytical uncertainty of ±0.02 units (about ±0.6°C; Prahl et al., 1988). Reproducibility of TEX_86_ is reported as ±0.004, corresponding to ±0.3°C (Schouten et al., 2007). We use 0.3°C as a cutoff value for the presence of lateral transport bias.

## 3 Results

Simulated trajectories show that travel distances are generally small (Table 1), with the mean across all sites for the slowest sinking speed (6 md^−1^) being 80 km and faster sinking speeds exhibiting smaller mean travel distances. Larger mean travel distances are associated with deeper water depths and slower sinking speeds since these factors prolong the time during which the particle sinks. Mean travel distance at an individual site reaches up to 293 km (HII‐H; 42.2°N, 3.8°E; Kim et al., 2015) for the 6 md^−1^ sinking speed. In general, sites in the western Mediterranean basin exhibit somewhat larger travel distances than those in the eastern Mediterranean after accounting for water depth (Figure S2 in Supporting Information S1).

**Table 1:** Summary Results of Lateral Transport Distance and Simulated Bias

	Travel distance (km)			Simulated lateral transport bias (°C)		
Simulation scenario	Mean	Maximum (site mean)	Maximum (trajectory)	Maximum magnitude of transport bias	Mean magnitude of transport bias	Count of sites with bias >0.3°C
30‐day surface	105	292	580	0.83	0.17	30
6 md^−1^ sinking speed	80	293	622	0.71	0.10	18
12 md^−1^ sinking speed	54	270	517	0.53	0.07	7
25 md^−1^ sinking speed	33	202	302	0.58	0.05	4
50 md^−1^ sinking speed	18	118	205	0.36	0.03	1
100 md^−1^ sinking speed	13	65	122	0.23	0.02	0
250 md^−1^ sinking speed	5	27	47	0.09	0.01	0
500 md^−1^ sinking speed	3	13	24	0.04	0.00	0

The 1000 md^−1^ trajectories show that this sinking speed is representative of conditions immediately above the burial site. The maximum mean travel distance for particles at this sinking speed is 7 km, well within the 1° coordinate box (about 111 km) often used for proxy calibrations, and within the resolution of the NEMO model itself (1/12°, about 9 km).

**Figure 2:**
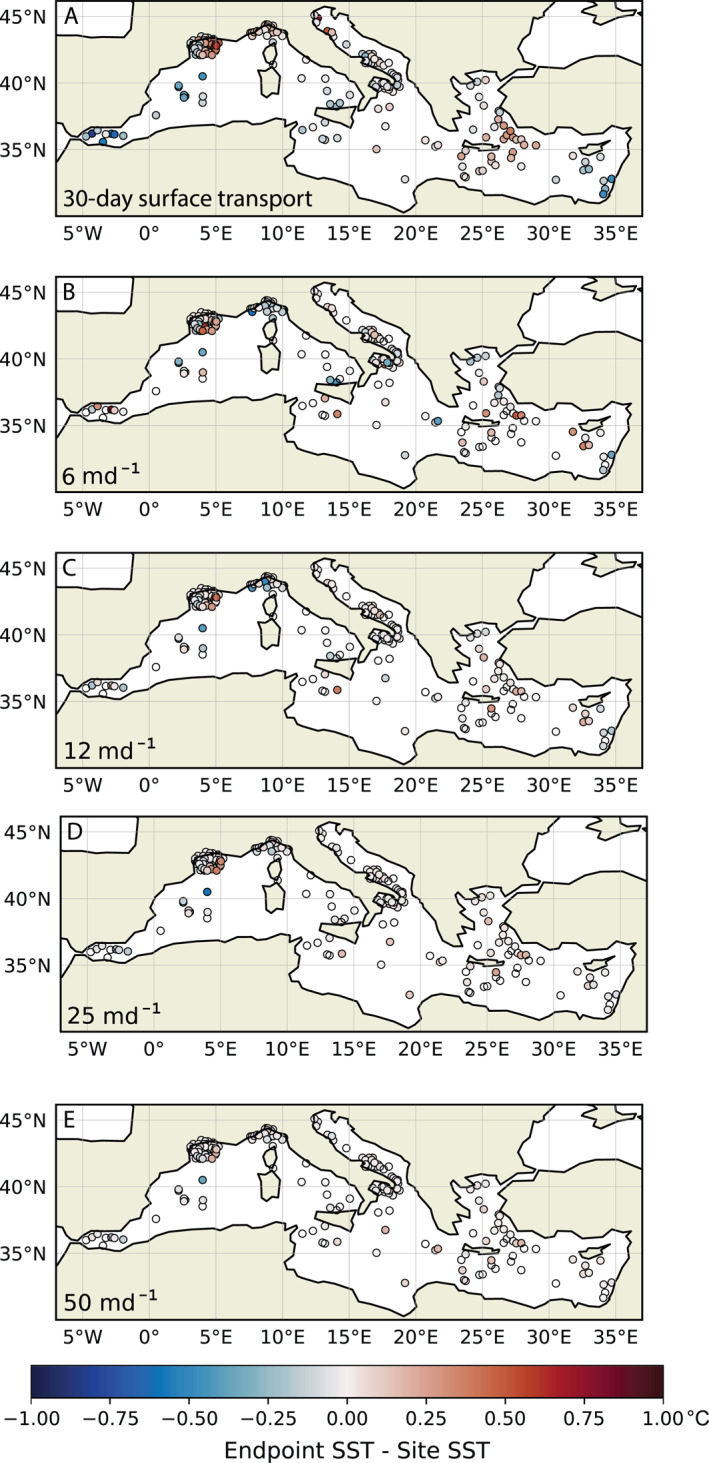
Lateral transport bias for (a) 30‐day surface advection and sinking speeds of (b) 6, (c) 12, (d) 25, and (e) 50 md^−1^, calculated as the difference between the mean sea surface temperature at trajectory endpoints and the mean at 1000 md^−1^ trajectory endpoints.

**Figure 3:**
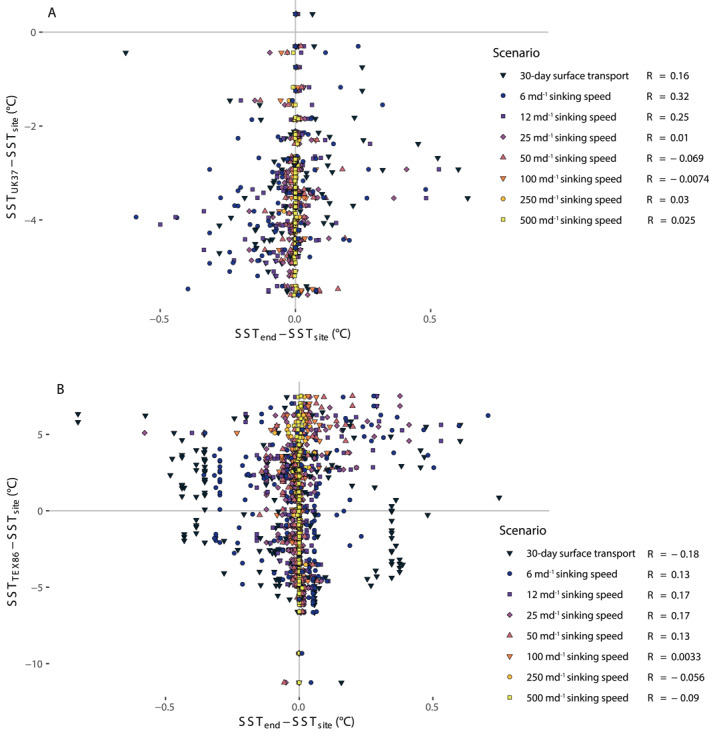
Proxy offset versus simulated lateral transport bias at surface sediment calibration sites for (a) U^K’^_37_ and (b) TEX_86_.

Mean simulated transport bias is negligible (up to 0.01°C across all scenarios), indicating that simulated trajectories come from areas with SSTs similar to the burial site, and/or from both warmer and colder waters. The mean transport bias is also small, reaching 0.1°C for the 6 md^−1^ sinking speed. Although most burial sites have a mean magnitude of transport bias near zero, certain individual sites have larger simulated transport bias, with a maximum magnitude of simulated transport bias of 0.7°C for the 6 md^−1^ sinking speed and 0.8°C for the 30‐day surface transport scenario (Figure 2). Transport bias exceeds the 0.3°C threshold at 30 surface sediment locations, less than 10% of the surface sediment sites. Simulated transport bias has no clear spatial distribution for the sinking scenarios. Transport biases in the 30‐day surface transport simulation do appear to vary by location, with cold biases in the Alboran Sea and western Mediterranean and warmer biases in and near the Aegean Sea. Simulated transport bias does not correlate with the proxy offset in U^K’^_37_ or TEX_86_ in any of the studied scenarios (Figure 3).

## 4 Discussion

### 4.1 Transport Bias During Alkenone Export

The range of simulated sinking speeds is appropriate for several proxy carriers. However, the results from each sinking speed alone may be a poor indicator of lateral transport bias. Wakeham et al. (2009) recorded a bimodal distribution of alkenone sinking speeds, with some alkenone‐carrying particles sinking at speeds between 11 and 49 md^−1^, and others at faster speeds, suggesting that a combination of several simulated sinking speeds is more appropriate than a single sinking speed to describe alkenone flux. Based on the sediment trap studies reviewed above, the 25 and 50 md^−1^ sinking speeds are the most appropriate to describe transport bias for the U^K’^_37_ paleothermometer. At these sinking speeds, lateral transport may contribute to proxy bias at up to four of the tested surface sediment sites.

Strong seasonality in alkenone production warrants an examination of seasonal transport bias. Tierney and Tingley (2018) show that U^K’^_37_‐based SSTs in Mediterranean core tops best correlate with November‐May SSTs, corresponding to winter‐spring maxima of alkenones and coccospheres observed in sediment trap studies (Malinverno et al., 2009; Skampa et al., 2020; Ternois et al., 1996; Triantaphyllou et al., 2004; Ziveri et al., 2000). Examining simulated trajectories where particles began sinking during these months (where the trajectory endpoint occurs during November–May) simulates transport of only seasonally produced alkenones. While seasonal SSTs reduce proxy bias, centering offsets close to zero, there is still no correlation with seasonal transport bias (Figure S3 in Supporting Information S1). Simulated seasonal transport bias also exhibits no clear spatial pattern and is small. However, the distribution of simulated transport bias is slightly skewed toward higher values (mean simulated transport bias across all sites of +0.1°C for the 6 md^−1^ sinking speed), indicating that simulated particles sinking between November‐May were more likely to originate in slightly warmer water than their burial site.

### 4.2 Transport Bias During GDGT Export

Because the sinking speed of GDGT‐carrying particles is poorly constrained, there is greater uncertainty in lateral transport bias. Several sediment trap studies note a lack of seasonal signal in TEX_86_ values (Chen et al., 2016; Fallet et al., 2011, 2012; Richey & Tierney, 2016), which may suggest that GDGT production does not respond to temperature changes on a seasonal scale, or that GDGTs produced during various seasons mix as they sink, implying slow sinking speeds. For example, particles sinking at a range of relatively slow sinking speeds between 6 and 25 md^−1^ would sink 1000 m in 40–167 days, obscuring seasonal changes in SST. GDGT production below the mixed layer could also result in a lack of seasonality (Huguet et al., 2007; Richey & Tierney, 2016). Despite local evidence for limited export of GDGTs below 100 m depth (Wuchter et al., 2005), several studies show contributions from deeper‐dwelling archaea to the sedimentary GDGT pool on a global scale (e.g., Ho & Laepple, 2016; Taylor et al., 2013; van der Weijst et al., 2021) and in the Mediterranean Sea (Kim et al., 2015). We focus our discussion on the 6 md^−1^ sinking speed departing from the mixed‐layer as a worst‐case scenario, but it is possible that even the 6 md^−1^ sinking speed does not adequately represent slow‐sinking GDGT‐carrying particles.

Transport bias does not correlate with proxy offsets in TEX_86_‐based SSTs (Figure 3). However, simulated transport distance does appear to be related to TEX_86_ proxy offset, a relationship not present for U^K’^_37_ (Figure S4 in Supporting Information S1), likely because both TEX_86_ values and simulated transport distance relate to water depth. Kim et al. (2015) note a strong correlation between TEX_86_ values and water depth in the Mediterranean and suggest that the TEX_86_ paleothermometer is only appropriate for sites with a water depth of at least 1000 m. After removal of shallow locations, simulated transport distance and TEX_86_ offset are not correlated (Figure S5 in Supporting Information S1). Given the production of GDGTs in intermediate water depths (Besseling et al., 2019; Kim et al., 2016), we examine how lateral transport may impact particles produced deeper in the water column. The location of the virtual particles were recorded at the 150 m water depth, which approximately corresponds to the top of the Levantine Intermediate Water, which hosts a deep population of Thaumarchaeota (Besseling et al., 2019). In most simulations, there is little difference between the endpoint locations for the 30 and 150 m water depth endpoints, with 75% of virtual particles with a 6 md^−1^ sinking speed traveling less than 51 km between 30 and 150 m water depth.

### 4.3 Spatial Variability in Transport

To investigate whether temperature offsets due to lateral transport bias have a consistent spatial variability in the Mediterranean Sea, the data set was binned by the subbasin (Figure S1 in Supporting Information S1) and examined for differences in transport distance (Figure S2 in Supporting Information S1) and lateral transport bias (Figure S6 in Supporting Information S1). While water depth is a controlling factor for transport distance and the magnitude of lateral transport bias, little difference is observed between subbasins. Still, for a given water depth somewhat less mean transport distance is observed in the eastern basins (Levantine and Ionian Seas) compared to the western Mediterranean and the Alboran Sea. Furthermore, sites in the western Mediterranean and Alboran Sea have the largest simulated transport bias. In the surface transport scenario, lateral transport bias appears to have more spatial variability, but this variability does not appear to relate to differences in proxy bias in the basin, suggesting that this scenario does not reflect proxy export. However, without a uniform spatial distribution of surface sediment sites, assessment of the spatial variability of lateral transport may be biased.

## 5 Conclusions

Although some particles travel long distances before burial in the Mediterranean Sea, the SST at the particle's origin is, on average, very similar to the SST at the burial site (<0.2°C offset), making lateral transport bias during sinking small and irrelevant to proxy reconstructions. Furthermore, the simulated bias introduced by lateral transport shows no relationship with bias in TEX_86_ and U^K’^_37_‐based SSTs in surface sediments, indicating that proxy uncertainty arises from factors other than lateral transport.

## Supporting information

Supporting Information S1

## Data Availability

Trajectory endpoint data and code to reproduce the results and figures in this paper are available at http://doi.org/10.5281/zenodo.6109228 (Rice, 2022).
