# Supplementary material for: Limited Lateral Transport Bias During Export of Sea Surface Temperature Proxy Carriers in the Mediterranean Sea
Source: Geophys Res Lett. 2022 Feb 23;49(4):e2021GL096859. doi: 10.1029/2021GL096859 (PMC9286692; doi:10.1029/2021GL096859)
Supplement: Supplementary file 1 — Supporting Information S1 [file GRL-49-0-s001.docx]

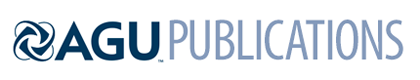


*Geophysical Research Letters*

Supporting Information for

**Limited lateral transport bias during export of sea surface temperature proxy carriers in the Mediterranean Sea**

Addison Rice^1^, Peter Nooteboom^2,3^, Erik van Sebille^2,3^, Francien Peterse^1^, Martin Ziegler^1^, Appy Sluijs^1^

^1^ Department of Earth Sciences, Utrecht University, Utrecht, The Netherlands

^2^ Utrecht University, IMAU, Department of Physics, Utrecht, Netherlands

^3^ Centre for Complex Systems Studies, Utrecht University, Utrecht, Netherlands

**Contents of this file**

Text S1

Figures S1 to S6

Text S1.

Several factors may contribute to offsets from expected values of U^K’^_37_ and TEX_86_ in the Mediterranean Sea, including seasonality (Sicre et al., 1999; Ternois et al., 1997; Tierney & Tingley, 2018), depth habitat (Besseling et al., 2019; Kim et al., 2015; Sicre et al., 1999; Ternois et al., 1997), and other environmental stressors (Prahl et al., 2003; Qin et al., 2015). In other parts of the globe, lateral advection of resuspended sediment has also been shown to influence U^K’^_37_ values by introducing allochthonous alkenones (Benthien & Müller, 2000; Fallet et al., 2012; Mollenhauer et al., 2007, 2008; Ohkouchi et al., 2002; Shah et al., 2008). This study focuses on the possible role of lateral transport during export on proxy values, however, a review of the literature on proxy bias in the region provides a necessary framework for interpreting our simulation results.

In the case of the U^K’^_37_ paleothermometer, much of the offset from expected values is attributed to seasonal (autumn-spring) blooms of alkenone-producing algae, which leads to U^K’^_37_ temperatures lower than the mean annual SST (Sicre et al., 1999; Ternois et al., 1997; Tierney & Tingley, 2018). However, U^K’^_37_ values have also been proposed to reflect temperature at or below the thermocline in the Mediterranean (Sicre et al., 1999; Ternois et al., 1997), and nutrient stress may also result in lower U^K’^_37_-based SST values (Prahl et al., 2003). Paleoclimate studies in the Mediterranean Sea often interpret U^K’^_37_ SSTs as winter values and downcore changes in U^K’^_37_ as variations in winter temperatures (Castañeda et al., 2010; Grauel et al., 2013; Versteegh et al., 2007). Interpretations can also involve changes in the intensity of vertical mixing (Grauel et al., 2013; Versteegh et al., 2007).

In addition, alkenones may be resuspended and laterally advected in nepheloid layers, particularly in continental margin sites (Benthien & Müller, 2000; Fallet et al., 2012; Mollenhauer et al., 2007, 2008; Ohkouchi et al., 2002; Shah et al., 2008). Off the coast of Namibia, this results in surface sediments that incorporate allochthonous, pre-aged particles thousands of years old, including alkenones exhibiting ^14^C ages up to 3490 years (Mollenhauer et al., 2008). However, a study of a sediment drift in the North Atlantic suggests little pre-aged alkenone transport, suggesting that this process is not important for proxy bias in all areas with resuspended sediment transport (Mollenhauer et al., 2011). Although alkenone ^14^C measurements have not been performed in the Mediterranean Sea, deep water cascades from the Gulf of Lions create thick bottom nepheloid layers in the Western Mediterranean basin, observed in 1999, 2005, and 2006 (Puig et al., 2013), which could result in U^K’^_37_ temperatures that incorporate allochthonous alkenones. In the Eastern Mediterranean, basin-wide bottom nepheloid layers have not been observed, and nepheloid layers instead appear to be restricted to coastal regions (Karageorgis et al., 2008). Transport after (initial) burial by resuspension and advection in bottom currents was not assessed in this study, but radiocarbon studies show that alkenones may be much older than GDGTs within the same sediment sample, indicating that GDGTs may degrade in the process of resuspension and burial while alkenones remain intact (Mollenhauer et al., 2007, 2008).

There has been much speculation on the underlying cause of high TEX_86_ -based SSTs in the Mediterranean Sea, with earlier studies suggesting that archaea thrived during summer conditions, resulting in a seasonal bias to warm values (Castañeda et al., 2010; Grauel et al., 2013; Huguet et al., 2011; Leider et al., 2010; Menzel et al., 2006; Nieto-Moreno et al., 2013). Some have also suggested that nutrient availability plays a role, with high-nutrient coastal sites exhibiting lower SSTs than those with open ocean conditions (Grauel et al., 2013; Leider et al., 2010). During sapropel formation, oceanographic changes may have triggered an adaptive response of their producers to low-oxygen conditions (Qin et al., 2015) or driven archaea to live at a different depth in the water column (Menzel et al., 2006; Polik et al., 2018). Water depth accounts for much of the variance in GDGT distributions in surface sediments in the Mediterranean, suggesting the presence of a population of deep-dwelling GDGT producers with a different membrane composition (Kim et al., 2015). Polik et al. (2018) also suggested that ecological shifts in the archaeal community could impact the isoGDGT distribution, creating anomalies in TEX_86_-based SSTs in restricted basins such as the Mediterranean Sea. Recently, Besseling et al. (2019), based on 16S rRNA gene copies and GDGT concentrations from suspended matter, found that TEX_86_ values overestimate SST due to differences in the archaeal community between the Mediterranean Sea and the global ocean. Specifically, Thaumarchaeota, or Marine Group I archaea, typically inhabit surface waters and produce most GDGTs in the global ocean. However, Marine Group I is not the dominant group in Mediterranean surface waters, which mostly consists of Marine Group II and III archaea. Furthermore, Mediterranean-specific Thaumarchaeotal OTUs occur in the surface water, which, combined with the contribution of deep water Thaumarchaeota, creates a different distribution of GDGTs in Mediterranean Sea sediments, leading to high TEX_86_ values (Besseling et al., 2019). Although the absolute temperature reconstructed by TEX_86_ has a strong warm bias, downcore variations in GDGT distributions in combination with other proxy results may still be useful as indicators of temperature or changing oceanographic conditions. Kim et al. (2015) found that TEX_86_ values and SST are still closely related when examining a dataset limited to surface sediments from over 1000 m water depth in the Mediterranean and Red Seas, and derived a region-specific calibration equation.


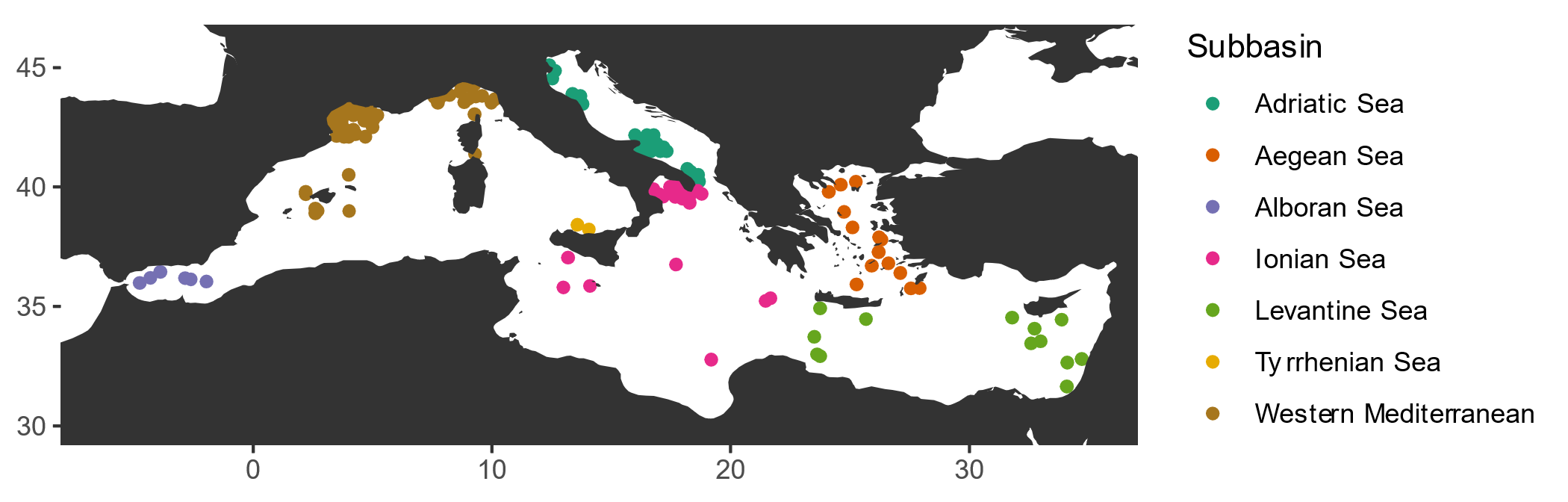


Figure S1. Surface sediment locations binned by subbasin.


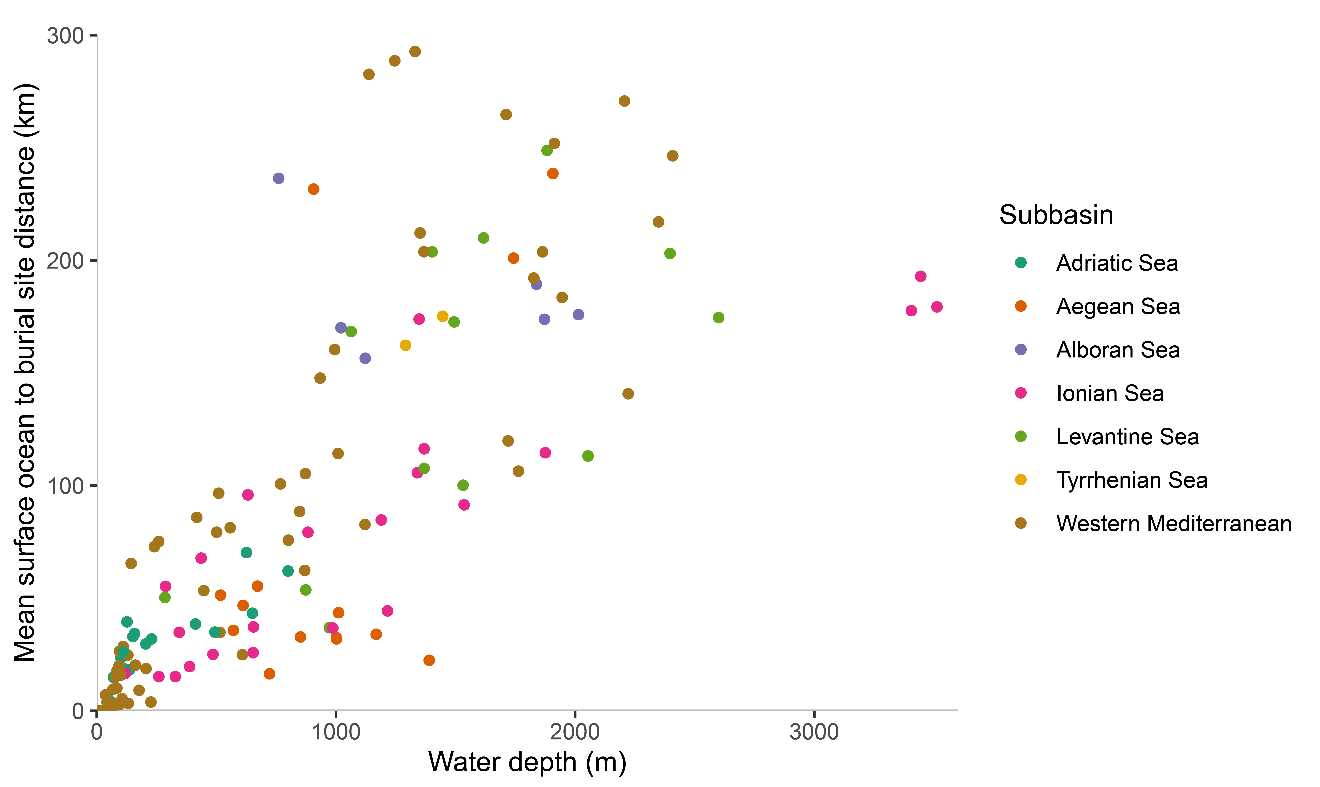


Figure S2. Water depth vs. distance from the burial site to the surface ocean for the 6 md^-1^ sinking speed. Colors refer to subbasins as in Figure S1.


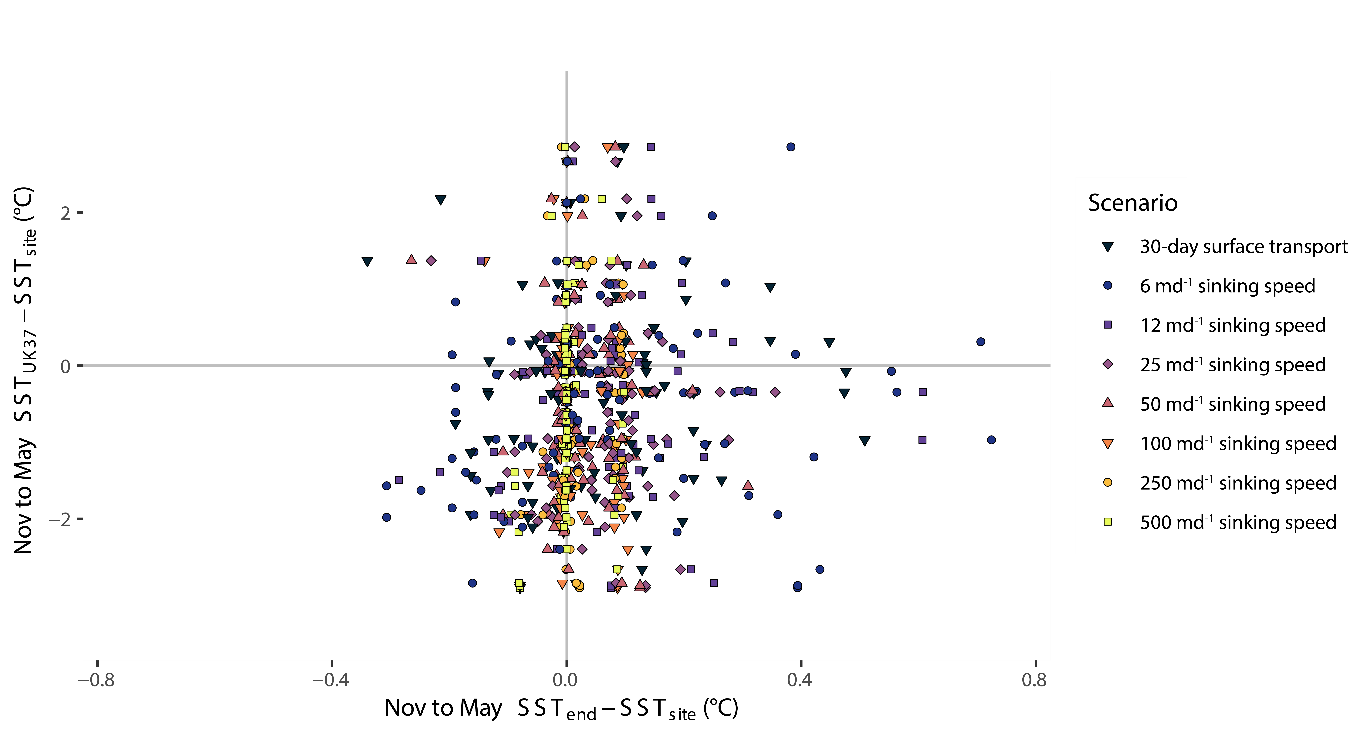


Figure S3. Simulated lateral transport bias using a seasonal average (trajectories reaching 30m water depth during Nov-May) vs U^K’^_37_ proxy offset from seasonal SSTs (SST_UK37_ –SST_Site, Nov-May_)


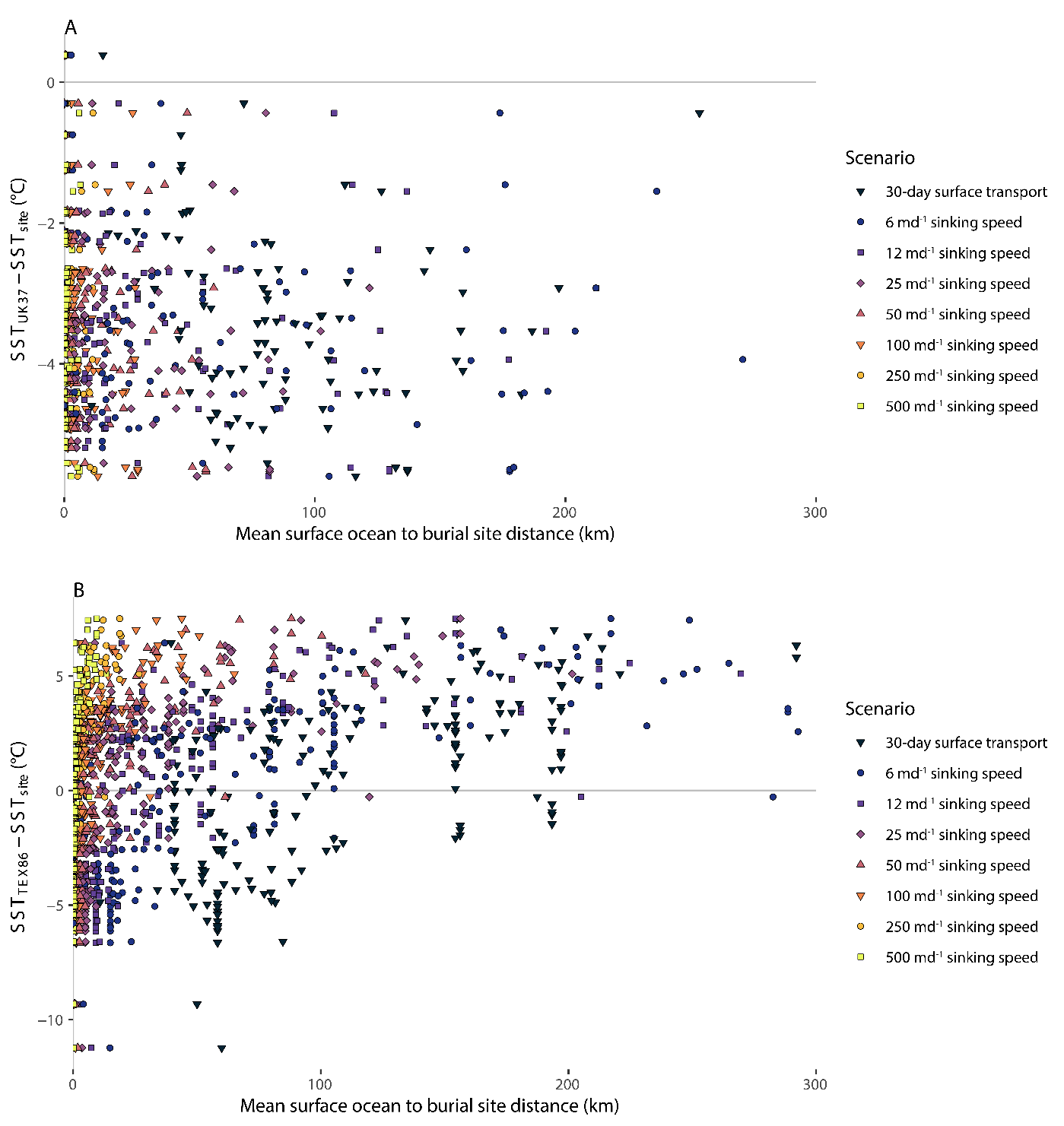


Figure S4. Simulated transport distance at (A) U^K’^_37_ and (B) TEX_86_ surface sediment sites vs proxy offset.


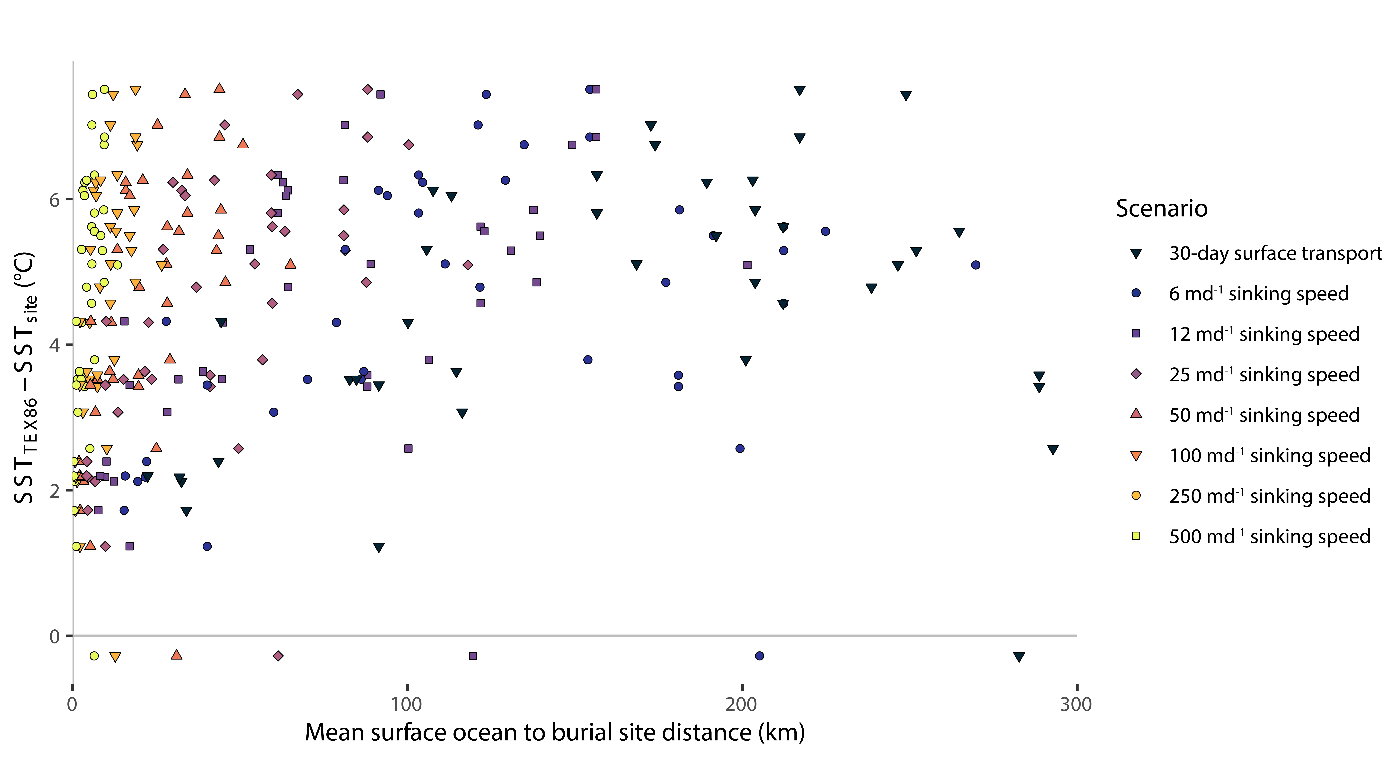


Figure S5. Simulated transport distance at TEX_86_ surface sediment sites >1000m water depth vs TEX_86_ proxy offset.


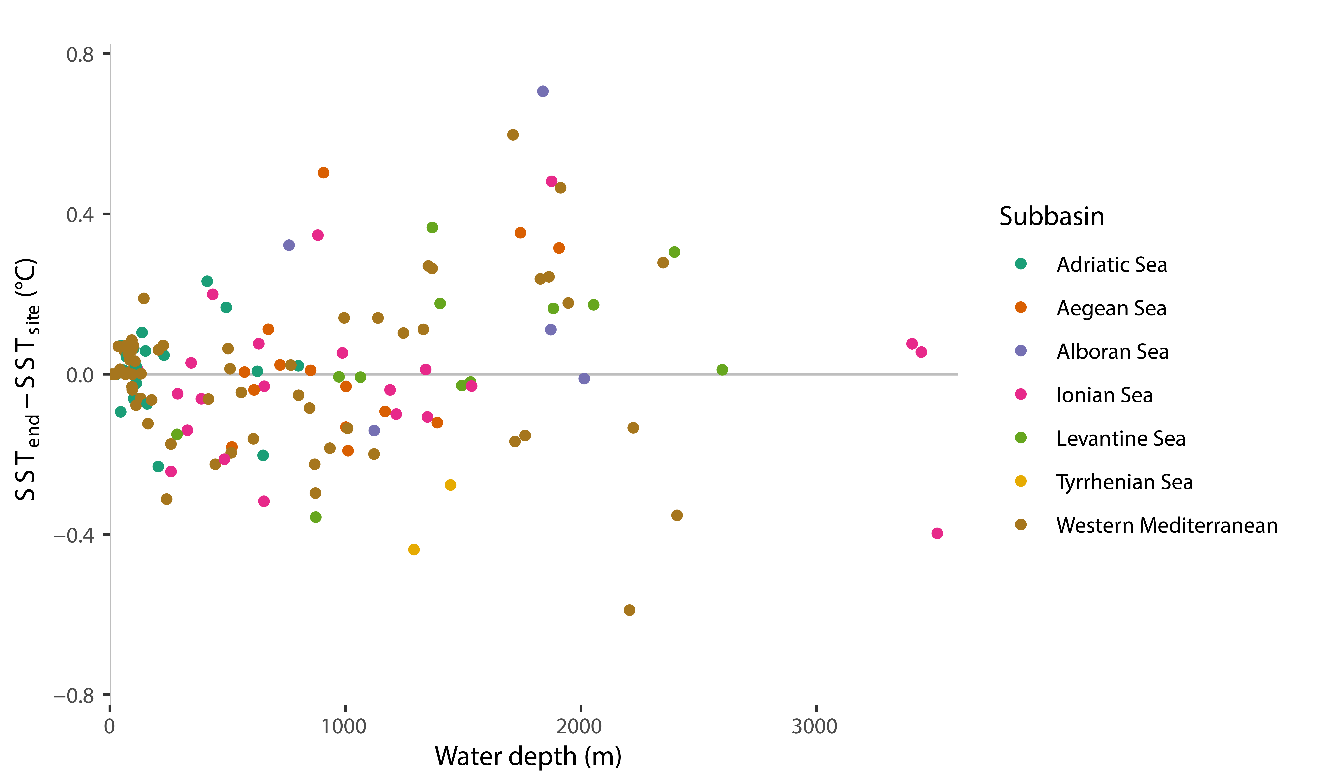


Figure S6. Water depth vs. lateral transport bias for the 6 md^-1^ sinking speed scenario. Colors refer to subbasins as in Figure S1.

References

Benthien, A., & Müller, P. J. (2000). Anomalously low alkenone temperatures caused by lateral particle and sediment transport in the Malvinas Current region, western Argentine Basin. *Deep-Sea Research Part I: Oceanographic Research Papers*, *47*(12), 2369–2393. https://doi.org/10.1016/S0967-0637(00)00030-3

Besseling, M. A., Hopmans, E. C., Koenen, M., van der Meer, M. T. J., Vreugdenhil, S., Schouten, S., et al. (2019). Depth-related differences in archaeal populations impact the isoprenoid tetraether lipid composition of the Mediterranean Sea water column. *Organic Geochemistry*, *135*, 16–31. https://doi.org/10.1016/j.orggeochem.2019.06.008

Castañeda, I. S., Schefuß, E., Pätzold, J., Sinninghe Damsté, J. S., Weldeab, S., & Schouten, S. (2010). Millennial-scale sea surface temperature changes in the eastern Mediterranean (Nile River Delta region) over the last 27,000 years. *Paleoceanography*, *25*(1), 1–13. https://doi.org/10.1029/2009PA001740

Fallet, U., Castañeda, I. S., Henry-Edwards, A., Richter, T. O., Boer, W., Schouten, S., & Brummer, G. J. (2012). Sedimentation and burial of organic and inorganic temperature proxies in the Mozambique Channel, SW Indian Ocean. *Deep-Sea Research Part I: Oceanographic Research Papers*, *59*, 37–53. https://doi.org/10.1016/j.dsr.2011.10.002

Grauel, A. L., Leider, A., Goudeau, M. L. S., Müller, I. A., Bernasconi, S. M., Hinrichs, K. U., et al. (2013). What do SST proxies really tell us? A high-resolution multiproxy (U^K'^_37_, TEX^H^_86_ and foraminifera δ^18^O) study in the Gulf of Taranto, central Mediterranean Sea. *Quaternary Science Reviews*, *73*, 115–131. https://doi.org/10.1016/j.quascirev.2013.05.007

Huguet, C., Martrat, B., Grimalt, J. O., Sinninghe Damsté, J. S., & Schouten, S. (2011). Coherent millennial-scale patterns in U^K'^_37_ and TEX^H^_86_ temperature records during the penultimate interglacial-to-glacial cycle in the western Mediterranean. *Paleoceanography*, *26*(2). https://doi.org/10.1029/2010PA002048

Karageorgis, A. P., Gardner, W. D., Georgopoulos, D., Mishonov, A. V., Krasakopoulou, E., & Anagnostou, C. (2008). Particle dynamics in the Eastern Mediterranean Sea: A synthesis based on light transmission, PMC, and POC archives (1991-2001). *Deep-Sea Research Part I: Oceanographic Research Papers*, *55*(2), 177–202. https://doi.org/10.1016/j.dsr.2007.11.002

Kim, J. H., Schouten, S., Rodrigo-Gámiz, M., Rampen, S., Marino, G., Huguet, C., et al. (2015). Influence of deep-water derived isoprenoid tetraether lipids on the TEX^H^_86_ paleothermometer in the Mediterranean Sea. *Geochimica et Cosmochimica Acta*, *150*, 125–141. https://doi.org/10.1016/j.gca.2014.11.017

Leider, A., Hinrichs, K. U., Mollenhauer, G., & Versteegh, G. J. M. (2010). Core-top calibration of the lipid-based U^K'^_37_ and TEX^H^_86_ temperature proxies on the southern Italian shelf (SW Adriatic Sea, Gulf of Taranto). *Earth and Planetary Science Letters*, *300*(1–2), 112–124. https://doi.org/10.1016/j.epsl.2010.09.042

Menzel, D., Hopmans, E. C., Schouten, S., & Sinninghe Damsté, J. S. (2006). Membrane tetraether lipids of planktonic Crenarchaeota in Pliocene sapropels of the eastern Mediterranean Sea. *Palaeogeography, Palaeoclimatology, Palaeoecology*, *239*(1–2), 1–15. https://doi.org/10.1016/j.palaeo.2006.01.002

Mollenhauer, G., Inthorn, M., Vogt, T., Zabel, M., Sinninghe Damsté, J. S., & Eglinton, T. I. (2007). Aging of marine organic matter during cross-shelf lateral transport in the Benguela upwelling system revealed by compound-specific radiocarbon dating. *Geochemistry, Geophysics, Geosystems*, *8*(9). https://doi.org/10.1029/2007GC001603

Mollenhauer, G., Eglinton, T. I., Hopmans, E. C., & Sinninghe Damsté, J. S. (2008). A radiocarbon-based assessment of the preservation characteristics of crenarchaeol and alkenones from continental margin sediments. *Organic Geochemistry*, *39*(8), 1039–1045. https://doi.org/10.1016/j.orggeochem.2008.02.006

Mollenhauer, G., McManus, J.F., Wagner, T., McCave, I.N., Eglinton, T.I. (2011). Radiocarbon and ^230^Th data reveal rapid redistribution and temporal changes in sediment focussing at a North Atlantic drift. *Earth and Planetary Science Letters 301*, 373–381. https://doi.org/10.1016/j.epsl.2010.11.022

Nieto-Moreno, V., Martínez-Ruiz, F., Willmott, V., García-Orellana, J., Masqué, P., & Sinninghe Damsté, J. S. (2013). Climate conditions in the westernmost Mediterranean over the last two millennia: An integrated biomarker approach. *Organic Geochemistry*, *55*, 1–10. https://doi.org/10.1016/j.orggeochem.2012.11.001

Ohkouchi, N., Eglinton, T. I., Keigwin, L. D., & Hayes, J. M. (2002). Spatial and temporal offsets between proxy records in a sediment drift. *Science*, *298*(5596), 1224–1227. https://doi.org/10.1126/science.1075287

Polik, C. A., Elling, F. J., & Pearson, A. (2018). Impacts of Paleoecology on the TEX_86_ Sea Surface Temperature Proxy in the Pliocene-Pleistocene Mediterranean Sea. *Paleoceanography and Paleoclimatology*, *33*(12), 1472–1489. https://doi.org/10.1029/2018PA003494

Prahl, F. G., Wolfe, G. V., & Sparrow, M. A. (2003). Physiological impacts on alkenone paleothermometry. *Paleoceanography*, *18*(2), 1–7. https://doi.org/10.1029/2002pa000803

Puig, P., Madron, X. D. de, Salat, J., Schroeder, K., Martín, J., Karageorgis, A. P., et al. (2013). Thick bottom nepheloid layers in the western Mediterranean generated by deep dense shelf water cascading. *Progress in Oceanography*, *111*, 1–23. https://doi.org/10.1016/j.pocean.2012.10.003

Qin, W., Carlson, L. T., Armbrust, E. V., Devol, A. H., Moffett, J. W., Stahl, D. A., & Ingalls, A. E. (2015). Confounding effects of oxygen and temperature on the TEX_86_ signature of marine Thaumarchaeota. *Proceedings of the National Academy of Sciences of the United States of America*, *112*(35), 10979–10984. https://doi.org/10.1073/pnas.1501568112

Shah, S. R., Mollenhauer, G., Ohkouchi, N., Eglinton, T. I., & Pearson, A. (2008). Origins of archaeal tetraether lipids in sediments: Insights from radiocarbon analysis. *Geochimica et Cosmochimica Acta*, *72*(18), 4577–4594. https://doi.org/10.1016/j.gca.2008.06.021

Sicre, M.-A., Ternois, Y., Miquel, J.-C., & Marty, J.-C. (1999). Alenones in the Northwestern Mediterranean Sea: interannual variability and vertical transfer. *Geophysical Research Letters*, *26*(12), 1735–1738. https://doi.org/10.1029/1999GL900353

Ternois, Y., Sicre, M. A., Boireau, A., Conte, M. H., & Eglinton, G. (1997). Evaluation of long-chain alkenones as paleo-temperature indicators in the Mediterranean Sea. *Deep-Sea Research Part I: Oceanographic Research Papers*, *44*(2), 271–286. https://doi.org/10.1016/S0967-0637(97)89915-3

Tierney, J. E., & Tingley, M. P. (2018). BAYSPLINE: A New Calibration for the Alkenone Paleothermometer. *Paleoceanography and Paleoclimatology*, *33*, 281–301. https://doi.org/10.1002/2017PA003201

Versteegh, G. J. M., De Leeuw, J. W., Taricco, C., & Romero, A. (2007). Temperature and productivity influences on U^K'^_37_ and their possible relation to solar forcing of the Mediterranean winter. *Geochemistry, Geophysics, Geosystems*, *8*(9), 1–14. https://doi.org/10.1029/2006GC001543
